# The findings of CT and MRI in patients with metanephric adenoma

**DOI:** 10.1186/s13000-016-0535-x

**Published:** 2016-10-27

**Authors:** Jing Yan, Jing-Liang Cheng, Chen-Fei Li, Yan-Bang Lian, Yuan Zheng, Xue-Ping Zhang, Chao-Yan Wang

**Affiliations:** 1 Department of MRI, the First Affiliated Hospital of Zhengzhou University, No.1 Jianshe East Rd, Erqi District, Zhengzhou, 450052 Henan Province China; 2 Department of Pathology, the First Affiliated Hospital of Zhengzhou University, Zhengzhou, 450052 China; 3 Department of Radiology, the First Affiliated Hospital of Zhengzhou University, Zhengzhou, 450052 China; 4 Operation Department, the First Affiliated Hospital of Zhengzhou University, Zhengzhou, 450052 China

**Keywords:** Metanephric adenoma, Computer tomography, Magnetic resonance imaging, Pathology

## Abstract

**Background:**

Metanephric adenoma (MA) is a benign renal tumor that is difficult to distinguish from a malignant tumor via traditional radiography. The diagnosis of MA is often dependent on postsurgical histopathological examination. In the present report, the imaging features of MA on computer tomography (CT) and magnetic resonance imaging (MRI) were retrospectively evaluated.

**Methods:**

Eight MA patients, 17–67 years of age, were pathologically confirmed and recruited between April 2009 and November 2014. Four of the eight patients were female. All patients underwent CT scanning, and one patient underwent MRI scanning. Three patients underwent CTA of the renal arteries. All patients underwent resection surgery (radical nephrectomy in five and nephron-sparing surgery in three patients).

**Results:**

The average tumor size was 44.0 ± 23.6 mm. The lesions in 87.5 % cases were located both in the renal cortex and medulla and exhibited exophytic growth. Plain CT showed that MA tumors were solid, and the average CT value was 37.9 ± 6.7 HU. Dynamic contrast-enhanced CT revealed that enhanced degrees of MA tumors in the renal cortex, renal parenchymal, and pelvic phase were all lower than that of normal renal parenchyma. A slight enhancement in the renal cortex phase and an even higher enhancement in the renal parenchymal phase were observed in seven of the cases. Progressive enhancement in the pelvic phase was found in five cases and a slight decreased enhancement in the pelvic phase in two cases. MRI revealed that MA tumor was isointense on T1WI and isointense on T2WI with some slightly hyperintense areas in the center. CTA of the renal arteries revealed the nutrient artery in one patient and no nutrient artery in two. Immunohistochemical experiments demonstrated that most tumor cells were positive for vimentin, CK, and EMA.

**Conclusions:**

MA is a rare benign renal neoplasm. Detailed knowledge of the CT and MRI characteristics of MA plays an important role in MA diagnosis and treatment.

## Background

Metanephric adenoma (MA) is described as a rare benign renal tumor presented at any age, especially in middle-aged people. It accounts for 0.2 % of adult renal epithelial tumors and is more common in females than in males [1]. Pathologically, MA arises from the residual renal tissue in the embryonic kidney development process. The pathologic morphogenetic characteristics and biological behavior of MA are unique [2]. It is easy to distinguish MA from the renal cell carcinoma. However, urologists are unable to differentiate MA from renal cell carcinoma through clinical or radiological checks [3, 4].

The clinical symptoms of MA include polycythemia, abdominal pain, hematuria, and a palpable mass [5, 6]. However, patients with MA are commonly asymptomatic and the lesions are incidentally found. The clinical and anatomic characteristics are not yet well defined for this rare type of renal tumor. There have been limited studies focusing on CT and MRI, and this has contributed to the low accuracy in diagnosing MA preoperatively using imaging. This has often resulted in un-necessary total nephrectomy.

Therefore, the purpose of this study was to assess the utility of CT and MRI in patients with MA. We retrospectively studied the CT and MRI features of eight pathologically-confirmed MA patients who were admitted to our center between April 2009 and November 2014.

## Materials and methods

### Patients

Eight MA patients diagnosed with pathology were included in this report. All patients were hospitalized at the First Affiliated Hospital of Zhengzhou University from April 2009 to November 2014. The detailed clinical parameters, clinical symptoms, and imaging features are displayed in Tables 1 and 2. This work was approved by the Ethics Committee of the First Affiliated Hospital of Zhengzhou University (reference number: 2013-No.5 speedy trial of scientific research). Written informed consent for publication of the patient’s information and images was obtained from all patients.

**Table 1:** The characteristics of subjects

Number	Gender	Age (years)	Symptoms	Treatment	Metastasis	Side	Tumor size (mm)	Shape
1	Female	28	Asthenia, asarcia, inappetence	RN	No	Left	38	Oval
2	Male	17	flank pain	RN	No	Right	94	irregular
3	Male	43	No	NSS	No	Right	40	Round
4	Female	28	No	NSS	No	Right	29	Round
5	Female	67	No	RN	No	Right	25	Round
6	Female	57	No	RN	No	Right	35	Round
7	Male	47	gross hematuria	RN	No	Right	24	Round
8	Male	60	flank pain	NSS	No	Right	35	Round

*RN* radical nephrectomy, *NSS* nephron-sparing surgery

**Table 2:** The CT and MRI characteristics of MA

Number	Calcification	Cystic changes or necrosis	Homogeneous/heterogeneous	plain CT (HU)	Enhanced CT			CTA	MRI
					cortex phase	parenchymal phase	pelvic phase		
1	No	No	Homogeneous	44	62	101	99	ND	ND
2	multi patchy	Multiple	Heterogeneous	44	109	153	89	Nutrient artery	ND
3	No	Yes	Slightly heterogeneous	44	52	57	51	No nutrient artery	ND
4	No	No	Homogeneous	41	51	58	70	ND	ND
5	No	No	Homogeneous	37	54	62	66	No nutrient artery	ND
6	No	Yes	Heterogeneous	25	45	65	78	ND	ND
7	No	No	Homogeneous	34	57	63	67	ND	ND
8	No	No	Homogeneous	34	54	60	71	ND	isointense on T1WI, isointense on T2WI with some slightly hyperintense areas in the center

*ND* not done

### CT scanning

All patients underwent CT scanning using a 64-slice CT scanner (LightSpeed VCT, GE Healthcare, USA). Plain CT and dynamic contrast-enhanced CT were performed. The scanning parameters were as follows: tube voltage, 120 kV; tube current, 250 mAs; detector collimation, 64 × 0.625 mm; gantry rotation time, 0.8 s/r; pitch, 0.984; and field-of-view, 250. The slice thickness was 5 mm in the plain scanning of bilateral kidneys. For contrast-enhanced CT scanning, 80–100 ml of omnipaque at a concentration of 350 mg/mL was injected into the antecubital vein. The injection dose was 1.4–1.6 ml/kg at a speed of 3.5 ml/s. Then, 30, 90, and 300 s after the injection of a contrast-enhancing agent, the patients went through the cortex phase, parenchymal phase, and pelvic phase. Three patients underwent CT angiography (CTA) of the renal arteries.

### MRI scanning

One patient underwent MRI scanning using a 3.0 T MR Scanner (MAGNETOM Verio, Siemens AG, Germany) with an eight-channel phased-array body coil. The following sequences were available for all the MR examinations: axial GRE T1-weighted in-phase/out-of-phase images (TR, 130 ms; TE, 4.8 ms and 2.5 ms respectively); axial FSE T2-weighted images with fat saturation (TR, 3000 ms; TE, 90 ms); flip angle, 70°; field of view, 40 × 40 cm; and matrix, 320 × 189. The patients performed a breath-hold in all the sequence scans mentioned above.

### Pathologic examination

All patients underwent tumor resection, five underwent radical nephrectomy, and three underwent nephron-sparing surgery. The shape and size of tumors were assessed via visual inspection. Cystic components and metastasis were evaluated during surgery. The tumor specimens were fixed with 10 % formaldehyde. Conventional paraffin sections were conducted subsequently. The histological and pathological results were assessed with hematein-eosin staining and immunohistochemical staining, respectively.

### Statistical analysis

All the data were analyzed using Microsoft Excel. The measurement data were shown as mean ± standard deviation (SD).

## Results

### Subjects’ characteristics and preoperative diagnosis

Eight patients were included in this report. The average age of patients was 43.4 ± 17.7 years (ranging from 17 to 67), with four females and four males. For the preoperative diagnosis, six cases were misdiagnosed as renal cell carcinoma, and two were misdiagnosed as renal hamartoma. Half of the patients had clinical symptoms (gross hematuria, *n* = 1; asthenia, asarcia, and inappetence, *n* = 1; flank pain, *n* = 2). The other four patients’ symptoms were discovered incidentally during physical examination.

### Characteristics of the tumors

As noted previously, all patients underwent tumor resection, five underwent radical nephrectomy, and three underwent nephron-sparing surgery. The average size of the tumors was 44.0 ± 23.6 mm (range from 24 to 94 mm). Only one was on the left side, and the other seven were on the right. Seven of the tumors were round or oval, and one was irregular in shape. Lesions were located both in the renal cortex and medulla in seven and projected outside of the renal contour. The tumor exhibited exophytic growth. However, the lesion was located in the medulla nephrica in only one patient.

### CT findings

**Fig. 1:**
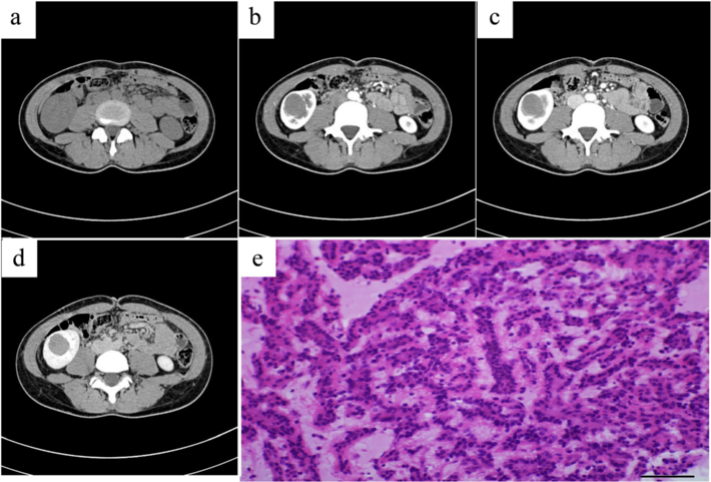
A 28-year-old female with metanephric adenoma in the mid and lower poles of the right kidney (case 4). Plain CT showed a round homogeneous isodense mass with a poorly defined margin in the renal medulla. The CT value of the mass was 41 HU (**a**). Dynamic contrast-enhanced CT revealed a progressive enhancement in the cortex phase (**b**), parenchymal phase (**c**), and pelvic phase (**d**). The CT values of all three phases, respectively, were 51 HU, 58 HU, and 70 HU, lower than that of the normal renal parenchyma. Pathology was assessed with hematein-eosin staining and showed that the morphology of tumor cells was uniform with tubular and acinar architecture (magnification, 40 × 10, **e**)

**Fig. 2:**
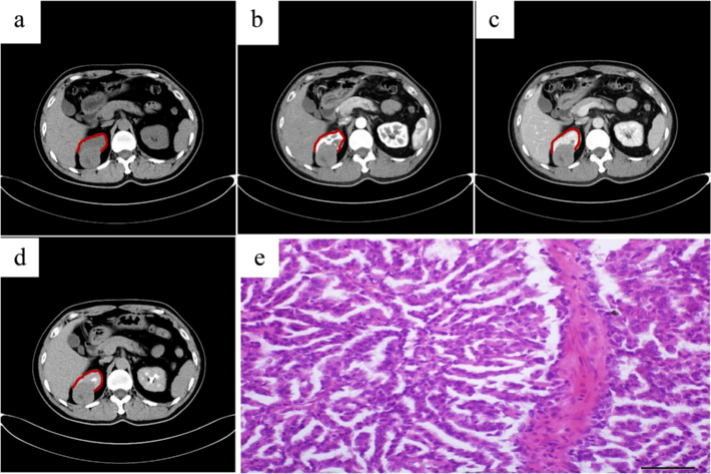
A 43-year-old male with metanephric adenoma in the upper pole of the right kidney (case 3). Plain CT showed a round, poorly defined isodense mass with a little patchy low-density areas in the renal cortex and medulla and the lesion projected outside of the renal contour. The CT value of the mass was 44 HU (**a**). Dynamic contrast-enhanced CT revealed a slight enhancement of the tumors in the cortex phase with a CT value of 52 HU (**b**), a further enhancement in the parenchymal phase with a CT value of 57 HU (**c**), and a slightly decreased enhancement in the pelvic phase with a CT value of 51 HU (**d**). Pathology was assessed with hematein-eosin staining and showed that the morphology of tumor cells was uniform with tubular and acinar architecture (magnification, 40 × 10, **e**). The kidney outline has been marked with red line

Plain CT showed that all the tumors were solid. The average CT value was 37.9 ± 6.7 HU. Seven of the tumors appeared as isodense (equal to the normal renal parenchyma, Figs. 1 and 2), and one as hypodense (lower than the normal renal parenchyma). The lesions were homogeneous in density in five patients (Fig. 1). Heterogeneous tumors were accompanied with few cystic changes or necrosis in two patients (Fig. 2) and with multiple patchy calcifications and cystic changes or necrosis in one case. Plain CT showed that the lesions were hardly distinguished from the normal renal parenchyma in all patients.

**Fig. 3:**
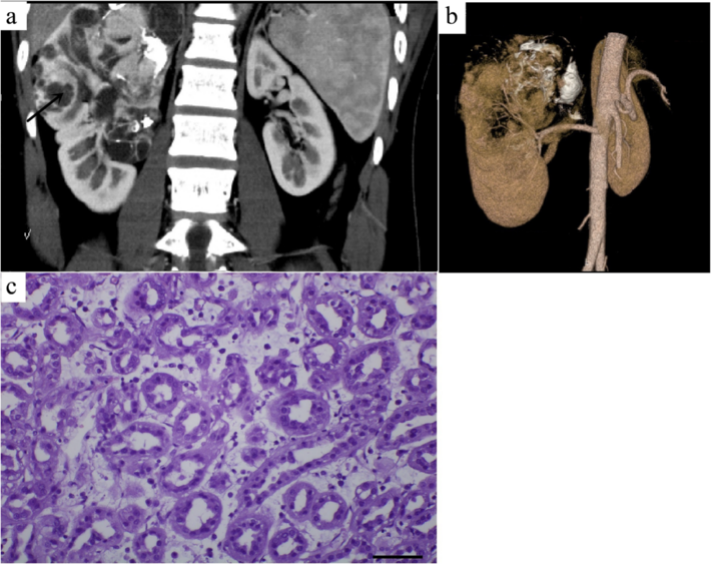
A 17-year-old male with metanephric adenoma in the mid and upper poles of the right kidney (case 2). Dynamic contrast-enhanced CT revealed an irregular lesion with a heterogeneous enhancement, multiple patchy calcifications, and cystic changes/necrosis (**a**). CT angiography revealed the nutrient artery in the lesion (**b**). Pathology was assessed with hematein-eosin staining and showed that the morphology of tumor cells was uniform with tubular and acinar architecture (magnification, 40 × 10, **c**). The arrow marker was used to indicate the cystic changes or necrosis

Dynamic contrast-enhanced CT revealed that enhanced degrees of the tumors in the renal cortex phase, renal parenchymal phase, and pelvic phase were all lower than that of the normal renal parenchyma (Figs. 1, 2, and 3). A slight enhancement in the renal cortex phase (Figs. 1b and 2b) and further enhancement in the renal parenchymal phase (Figs. 1c and 2c) were observed in seven patients. Progressive enhancement in the pelvic phase (Fig. 1d) was observed in five patients and a slight decreased enhancement in the pelvic phase in two (Fig. 2d). One patient had a phyma with irregular mixed density, and the enhanced CT showed a heterogeneous enhancement (Fig. 3a). Solid parts were obvious enhancements in the renal cortical phase, continuous enhancements in the parenchymal phase, and obvious decreased enhancements in the pelvic phase. In different phases of the enhanced CT, the lesions were distinguished from the normal renal parenchyma. Three patients underwent CTA of the renal arteries; the images revealed the nutrient artery in one patient (Fig. 3b) and no nutrient artery in two.

### MRI findings

**Fig. 4:**
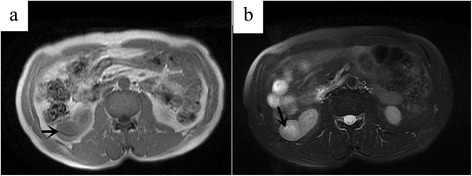
A 60-year-old male with metanephric adenoma in the lower pole of the right kidney (case 8). T1WI of the tumor showed nearly isointense to renal parenchyma (**a**). T2 weighted, fat suppressed image showed nearly isointense to renal parenchyma with some slightly hyperintense areas in the center (**b**). The arrow marker was used to indicate the lesion

One patient underwent MRI scanning. T1WI of the tumor showed nearly isointense to renal parenchyma (Fig. 4a). T2 weighted, fat suppressed image showed nearly isointense to renal parenchyma with some slightly hyperintense areas in the center (Fig. 4b).

### Metastasis and hydronephrosis

In this report, no metastasis of enlarged retroperitoneal lymph nodes, renal vessels, and the inferior vena cava was observed in any case. No patients had hydronephrosis.

### Pathological characteristics

**Fig. 5:**
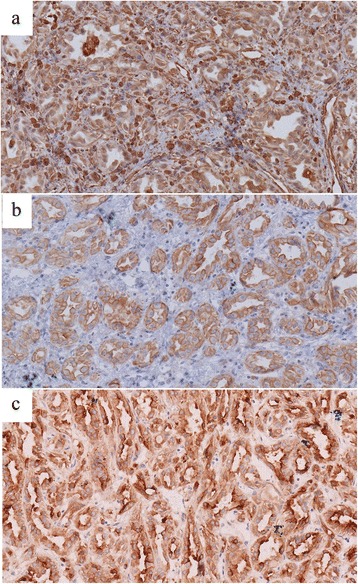
Immunohistochemical images for vimentin, CK and EMA. Immunohistochemical experiments revealed that most tumor cells were positive for vimentin (magnification: 200×, **a**), CK (magnification: 200×, **b**) and EMA (magnification: 200×, **c**)

The pathological characteristics of MA are shown in Figs. 1e, 2e, and 3c. Solid neoplasia was observed in the kidney and easily distinguished with adjacent tissue via visual inspection. The sections of tumors were shown in gray. The oncocytes were isolated and examined using a microscope. The results demonstrated that the cell morphology was uniform, with non-prominent nucleoli and little eosinophilic cytoplasm. In addition, tumor cells showed tubular and acinar architecture, leading to the formation of glomerular-like or bud-like structures. Intercellular substances showed acellular edema, myxoid, and hyaline degeneration. Immunohistochemical experiments revealed that most tumor cells were positive for vimentin, CK, and EMA, which confirmed the diagnosis of MA (Fig. 5).

## Discussion

MA is the occurrence of an uncommon renal tumor and was first described as a bilateral and diffuse tumor by Bove et al. in 1979 [7]. The histologic origin of MA remains controversial, but most scholars consider it to be derived from metanephric blastema. In recent years, emerging MA cases have been reported. MA generally occurs in adults, with a peak age of occurrence in the fifth or sixth decade of life, but it is also diagnosed in children [8, 9]. In the present study, patients’ average age and the age range at diagnosis was 43.4 years and 17–67 years. It is worth noting that three cases were under 30 years of age. Unilateral renal onset is more common, and all our patients were taken bad for unilateral kidney. However, there are also bilateral renal cases in a previous report [10]. In addition, MA seems to be more common in women than in men [2]. Nevertheless, no gender difference was observed in this report due to the small sample size.

As was the case with our patients, 50 % were clinically asymptomatic and diagnosed during physical examination [2]. The symptomatic cases of MA manifested as gross hematuria and flank pain in this study. Other symptoms, including polycythemia, a palpable mass, backache, abdominal pain, and fever, have been frequently presented in previous reports [11]. It has been also demonstrated that some patients have symptoms of urinary tract infections (UTIs). MA has been reported to have the highest level of polycythemia among all kidney tumors [2], which is probably related to the production of erythropoietin and multiple cytokines by MA [12]. Asymptomatic cases go against the discovery of MA. Therefore, physical examination is important for detection in early stages.

The clinical and imaging features of MA are complex and varied. It is difficult to give a final conclusion through preoperative diagnosis, due to the fact that it is often misdiagnosed as renal cell carcinomas, renal cysts, and other kidney diseases [3, 13]. In this report, six patients were preoperatively misdiagnosed as renal cell carcinoma and two were preoperatively misdiagnosed as renal hamartoma. Currently, the final diagnosis has to rely on pathology [3]. CT is the main imaging method for the diagnosis of MA. However, no typical radiological features of MA have been identified [14]. In this study, plain CT showed that all MA tumors were solid and 87.5 % of the lesions were identified to be isodense and equal to the normal renal parenchyma. Dynamic contrast-enhanced CT is the principal characteristic of MA, and it revealed that enhanced degrees of the tumors in the renal cortex, renal parenchymal, and pelvic phase were mainly progressive and the levels in all three phases were lower than that of the normal renal parenchyma. MA lacks a blood supply, and the nutrient artery and neoangiogenesis are rarely observed in lesions. However, we observed the nutrient artery in one patient. The features described above help to identify MA and renal clear cell carcinoma. Renal clear cell carcinoma is the most common tumor in the kidneys with an abundant blood supply and the enhancement pattern of rapid rise-rapid fall, and the enhanced degree of this tumor is generally higher than that of the normal renal parenchyma. Calcification is considered a critical indicator for the diagnosis of MA [15]. Only one of the eight MA tumors showed calcification in this report. In addition, cystic changes and necrosis were confirmed in 37.5 % of MA tumors. We found that MA lesions were located both in the renal cortex and medulla in 87.5 % of patients. Zhu et al. reported that seven of eight MA tumors were centered in the renal medulla [16]. However, a recent study shows that only 16.7 % of tumors were located entirely within the renal parenchyma and 83.3 % were located at the periphery of the renal cortex without involvement of the renal collecting system [17]. In a previous report, the renal cortex has been indicated as the predilection site of MA [2]. The sample size greatly contributes to the conflicting CT findings for MA. In addition, we found that the lesions projected outside of the renal contour exhibited exophytic growth, which is one of the principal characters of MA.

The MRI findings of MA are relatively limited. The typical MRI finding of MA is hypointense (or isointense) on T1WI and T2WI [4, 11]. In our report, one case of MA was isointense on T1WI and isointense on T2WI with some slightly hyperintense areas in the center. Due to the diversification of MRI findings, more cases need to be collected and analyzed.

Rare metastatic MA has been reported. In addition, metastasis of the retroperitoneal enlarged lymph nodes, renal vessels, and the inferior vena cava was not observed in any case. However, lung metastasis and lymph node metastasis have been found in a few MA patients [18, 19]. A follow-up study is important for verifying metastatic MA.

The histological features of MA are distinctive and are characterized by epithelial cells with different amounts of cytoplasm. The adenomatous components are composed of uniform small cells arranged in tubular or papillary architectural patterns [11]. In the present patients, immunohistochemical staining showed that neoplastic cells were positive for vimentin, CK, and EMA. Previous findings demonstrate that CD57, AE1/AE3, and CAM5.2 are also positive in MA, while NSE, CEA, CgA, Syn, actin, desmin, and AMACR are negative [3, 20]. Pathological detection remains the most effective method for MA diagnosis.

## Conclusions

MA is a rare benign renal neoplasm at any age. It is easily misdiagnosed in preoperative diagnosis. CT and MRI findings assist clinicians to better discover and detect MA. It is even more important to avoid unnecessary total nephrectomy. However, the final diagnosis relies on pathology.

## Abbreviations

CT: Computer tomography
CTA: CT angiography
MA: Metanephric adenoma
MRI: Magnetic resonance imaging
SD: Standard deviation
UTI: Urinary tract infection
